# Understanding Atopic Dermatitis: Pathophysiology and Management Strategies

**DOI:** 10.3390/biom15111500

**Published:** 2025-10-24

**Authors:** Heng Chai, Wing Sum Siu, Hui Ma, Yuzhen Li

**Affiliations:** 1 Department of Dermatology and Venereology, The Second Affiliated Hospital of Harbin Medical University, Harbin 150081, China; 202301443@hrbmu.edu.cn; 2 Institute of Chinese Medicine, The Chinese University of Hong Kong, Shatin, New Territories, Hong Kong SAR, China; sammysiu@cuhk.edu.hk; 3 Guangdong-Hong Kong-Macau Joint Laboratory for Pharmacodynamic Constituents of TCM and New Drugs Research, The Chinese University of Hong Kong, Shatin, New Territories, Hong Kong SAR, China

**Keywords:** atopic dermatitis, pathophysiology, management

## Abstract

Atopic dermatitis (AD) is a chronic inflammatory skin condition characterized by itching, redness, and dryness, significantly impacting the quality of life of affected individuals. With a rising prevalence across diverse demographics, understanding AD is crucial due to its systemic nature and association with comorbidities such as asthma and allergic rhinitis, as well as its psychosocial implications. The pathophysiology of AD involves a complex interplay of genetic predispositions and environmental triggers, leading to dysbiosis and increased susceptibility to superinfection. Clinically, AD manifests variably across age groups, with distinct presentations in pediatric and adult populations. Diagnosis is primarily based on clinical assessment criteria, supplemented by differential diagnoses and, when necessary, skin tests for allergies. Current management strategies encompass topical therapies, including moisturizers, corticosteroids, and calcineurin inhibitors, alongside systemic treatments such as antihistamines, immunosuppressants, and biologics. Lifestyle modifications, including trigger avoidance and effective skin care routines, are essential components of comprehensive care. Emerging novel therapies targeting specific biomarkers are currently under investigation in clinical trials, offering promising avenues for more effective management. However, challenges remain in optimizing treatment protocols and addressing the multifaceted nature of AD. In conclusion, this review highlights the need for continued research and awareness regarding atopic dermatitis. A multidisciplinary approach to management is essential to enhance patient outcomes and address the complexities of this prevalent and impactful condition.

## 1. Introduction

Atopic dermatitis (AD), often referred to as eczema or atopic eczema, is recognized as the most prevalent chronic inflammatory skin condition. This disorder is primarily characterized by intense itching and skin inflammation, which can severely affect the quality of life for both patients and their families. The impact of AD extends beyond physical symptoms, influencing emotional and social well-being [1]. AD can develop at any age; however, approximately 60% of individuals affected by the condition experience its onset within the first year. The highest incidence is typically observed between the ages of 3 and 6 months [2]. Current estimates indicate that the global prevalence of AD stands at around 2.6%, affecting approximately 204.05 million people worldwide [3]. As a leading contributor to the global burden of skin diseases, the persistent and often severe symptoms of AD can lead to significant psychosocial challenges. Patients frequently face increased risks of comorbid conditions, including attention deficit/hyperactivity disorder, other atopic diseases, and various mental health disorders. The interplay of these factors can create a complex web of challenges that extend beyond the individual, affecting family dynamics and overall quality of life. Moreover, the economic burden associated with AD is substantial. In the United States alone, the annual costs related to the condition are estimated to exceed $5 billion, encompassing expenses for medications, hospitalizations, transportation, and adjustments in careers [4]. Given the profound impact of AD on patients’ lives, including its associated comorbidities, psychosocial effects, and financial implications, there is a pressing need for deeper insights into the condition’s underlying pathophysiology. Additionally, the development of innovative treatment options is important to improve clinical outcomes and alleviate the burden of this widespread skin disorder.

## 2. Pathophysiology of AD

**Figure 1:**
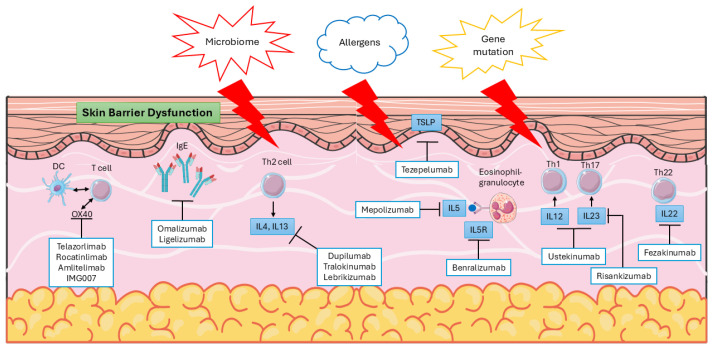
Pathophysiology and targeted therapy for AD.

Not only genetic factors, but also environmental elements are recognized as significant risk factors for AD occurrence. These factors contribute to skin barrier dysfunction and impair cell-mediated immune responses. Dysfunction of the normal skin barrier is one of the primary pathophysiological changes observed in AD, and it can arise from genetic mutations, cell-mediated processes, and other contributing factors. This dysfunction may drive the progression of the disease (Figure 1).

Numerous genetic mutations have been associated with AD, among which the filaggrin gene (FLG) mutation is the most extensively studied. First reported to have a strong correlation with AD in 2006, this mutation leads to reduced levels of filaggrin protein, which compromises the epidermal barrier. This impairment results in increased trans-epidermal water loss, alterations in skin pH, and dehydration [5]. It is important to mention that diminished filaggrin levels have been noted in AD patients with the wild-type genotype for FLG. This suggests that FLG variants are not the only factors responsible for the downregulation of filaggrin in the skin of individuals with AD. Additional elements, such as environmental and metabolic factors, are likely implicated as well [6]. In addition to FLG, several other genes associated with epidermal barrier function have been implicated in the development of AD. These include genes related to corneodesmosomes (such as desmoglein and desmocollin), tight junctions (claudins and occludins), and epidermal proteases (kallikreins, cathepsins, and caspase 14), as well as their inhibitors (such as spinous layer protein 5 (SPINK5) and Cystatin A). The transcription factor ovo like transcriptional repressor (OVOL)1, which regulates FLG expression, is also involved [7]. Years ago, genome-wide association studies (GWAS), a powerful method for identifying disease susceptibility genes associated with common human diseases, began to reveal underlying cellular pathways and point to new therapeutic approaches in AD [8]. A recent large-scale GWAS on AD, with a discovery cohort of 1,086,394 participants and a replication cohort of 3,604,027 participants, identified 81 loci (29 of which were novel) in the European-only analysis. Additionally, 10 more loci (3 novel) were identified in the multi-ancestry analysis [9]. The functions of the identified loci are associated with the positive regulation of T cell differentiation, development of hematopoietic or lymphoid organs, regulation of cytokine production and leukocyte proliferation, and positive regulation of protein and DNA metabolic processes. They also influence DNA-binding transcription factor activity and receptor signaling pathways via the JAK-STAT pathway [9]. Tissue enrichment analyses indicated that the strongest enrichment (odds ratio [OR] > 5.5 and *p* < 1 × 10^−10^) was observed in T cells, B cells, and natural killer lymphocytes (CD3+, CD4+, CD56+, and CD19+). As expected in AD, Th2 cells showed stronger enrichment (OR = 4.3, *p* = 1 × 10^−8^) compared to Th1 cells (OR = 2.3, *p* = 2 × 10^−4^) [9]. These findings correspond to the biologics and small molecules currently in use, which will be discussed in a later session. Based on the evidence from GWAS gene prioritization methods, the top prioritized gene is located on chromosome 12, specifically RPS26 and SUOX. The RPS26 gene encodes a ribosomal protein essential for ribosome assembly, serving as a component of the small subunit of the ribosome, thereby playing a fundamental role in protein synthesis. While the precise biological functions of RPS26 remain incompletely understood, many studies have focused on its association with various diseases and its potential as a biomarker, including Diamond-Blackfan anemia (DBA), type 1 diabetes, and fragile X-associated conditions [10,11,12]. In a study concerning attention deficit hyperactivity disorder (ADHD), single-cell sequencing of white blood cells revealed that RPS26 is related to monocyte apoptosis and proliferation. When RPS26 was knocked down, it impaired monocyte-to-macrophage maturation and altered the ratio of 28S to 18S RNA, indicating that RPS26 plays a regulatory role in monocyte development and differentiation [13]. Macrophages, the differentiated form of monocytes, play a critical role in inflammation, a key factor in AD, suggesting that RPS26 could serve as a potential biomarker and therapeutic target in treating AD. SUOX (sulfite oxidase) is an enzyme that plays a crucial role in the metabolism of sulfur-containing compounds, specifically in the conversion of sulfite to sulfate. Its activity is closely linked to oxidative stress. One study has investigated the correlation between SUOX and diabetes, focusing on the underlying mechanisms of oxidative stress [14]. However, there are currently no studies examining the relationship between SUOX and AD. Given that oxidative stress can impair the normal function of the skin barrier and considering the established connection between SUOX and oxidative stress, it is reasonable to propose that SUOX may serve as a potential target for strategies aimed at protecting the skin barrier and enhancing recovery in AD.

Cell-mediated skin barrier dysfunction is triggered in response to invading allergens and antigens. In the skin affected by atopic dermatitis, there is an increase in dendritic cells, which possess IgE receptors that facilitate antigen uptake and bolster cutaneous T-cell responses [15]. Alongside keratinocytes, these cells release inflammatory cytokines, including thymic stromal lymphopoietin (TSLP) and T-lymphocyte-associated protein 4 (TLA4), as well as chemokines that attract other immune cells, such as B cells, eosinophils, and various T cell subtypes [16]. Multiple T cell subtypes, including T helper (Th)1, Th2, Th17, and Th22 cells, are activated in response to this immune initiation. Among these, Th2 cells are particularly prominent in mediating type 2 inflammation through the secretion of cytokines such as interleukins (IL)-4, IL-5, IL-13, and IL-31 [17]. The activation of Th1 cells is supported by pro-inflammatory cytokines, including IL-1α, interferon (IFN)-α, and IFN-γ, released by skin dendritic cells during chronic AD [18]. Th22 cells contribute to skin remodeling and thickness through the secretion of IL-22 [17,18]. The role of B lymphocytes in AD remains unclear, despite the observed expansion of the B-cell lineage in the bloodstream of affected individuals [19]. Additionally, skin barrier dysfunction may also arise as a secondary consequence of primary immune defects, wherein the initial immune dysregulation triggers skin inflammation, leading to subsequent abnormalities in the epidermal barrier, microbial dysbiosis, and allergen penetration [20]. In the context of single-cell analysis, spatial transcriptomics sequencing can be employed to identify cellular infiltrates in AD. A study utilizing suction blister material has uncovered novel cellular crosstalk within leukocyte-infiltrated lesions associated with AD. This investigation has identified specific populations of fibroblasts, dendritic cells, and macrophages, including COL18A1-expressing fibroblasts, CCR7-expressing dendritic cells (DCs), and M2 macrophages expressing CCL13 and CCL18. Furthermore, ligand-receptor interaction analysis of the spatial transcriptome has elucidated their interactions [21]. Nevertheless, the precise relationship between their functions remains to be fully clarified. This study enhances our understanding of the pathophysiology of AD and highlights potential therapeutic targets for its treatment.

Multiple environmental factors contribute to the increased incidence of AD, including pollution, climatic conditions, and differences between rural and urban environments. Air pollution, especially in the form of airborne particulate matter (PM), is recognized for its role in disrupting the skin barrier and facilitating the production of reactive oxygen species, which contributes to oxidative stress. This process can lead to epigenetic alterations and skin inflammation via both direct and indirect pathways [22]. Prolonged exposure to low environmental humidity further exacerbates trans-epidermal water loss in individuals with AD, intensifying barrier defects and enhancing cytokine signaling of inflammatory molecules [12]. The “hygiene hypothesis” posits that greater microbial exposure during early life can prevent the development of AD later on [23], which may help explain the higher incidence of AD observed in urban areas. Additionally, external psychological stressors can also contribute to the skin barrier issues commonly seen in AD [24].

Currently, researchers agree that the pathophysiology of complex diseases is driven by the interaction between genetic and environmental factors. The variability in risk and outcomes in these diseases is incompletely explained by genetics or environmental risk factors individually. Therefore, researchers are now exploring the epigenome, a biological interface at which genetics and the environment can interact. Epigenome-wide association studies (EWAS) investigate the association between a phenotype and epigenetic variants, most commonly DNA methylation [25]. DNA methyltransferase-1 (DNMT-1), one of the related enzymes, a study has demonstrated the correlation of its level and AD severity. It has been reported that DNMT1-low AD patients exhibited a higher itch score compared to AD patients with high DNMT1 expression [26], making it a potential marker for disease severity. There is limited EWAS on AD published. There is one report demonstrating epigenetic influence at CpG site cg13920460 is associated with rs7872806 located deep intronic at 9q21.11. The single-nucleotide polymorphism (SNP) is related to AD by altering tight junction protein 2 expression and promoting trans-epidermal water loss [27].

The candidate loci identified through GWAS and EWAS only correlate genetically predicted expression with disease risk at the messenger RNA level, overlooking protein abundance and thus failing to establish direct connections with drug targets. In response to this limitation, proteome-wide association studies (PWAS) have emerged as a novel protein-based approach for identifying proteins associated with specific diseases or phenotypes [28]. A PWAS investigation has identified five proteins—IL18R1, MMP12, TAP-BPL, TLR1, and MFNG—as playing significant roles in the pathophysiology of AD [29]. Upon reviewing related drugs in clinical trials via www.clinicaltrials.gov, we found that Zileuton, which targets MMP12 [29], is currently being investigated in clinical trials for asthma as an anti-inflammatory agent (NCT00534625, NCT00575861, NCT00486343, NCT00299065). However, there are no registered trials for Zileuton in the context of AD. Given its anti-inflammatory properties, Zileuton has great potential to be developed as a new therapy for AD.

The change in microbiome in AD patients, including gut and skin microbiome, is related to the disease severity. The gut-skin axis, which represents the bidirectional communication between the gut microbiome and the skin, has been investigated as a potential contributor to the pathogenesis of AD. Dysregulation in the composition and function of the gut microbiome has been linked to the development and severity of AD [30]. Studies have shown alterations in microbial diversity among AD patients, including an increased prevalence of *Clostridium difficile*, *Escherichia coli*, and *Staphylococcus aureus* compared to healthy individuals. In contrast, AD patients exhibit reduced colonization of beneficial bacteria such as *Bifidobacteria*, *Bacteroidetes*, and *Bacteroides* compared to healthy controls [31]. Consequently, modulation of the gut microbiota through dietary adjustments, prebiotics, probiotics, and fecal microbiota transplantation has emerged as a promising strategy for the management of AD. Dysregulation of the skin microbiome, referred to as skin dysbiosis, may serve as a predictor of the severity of AD. An increased load of *Staphylococcus aureus* has been observed in AD patients, associated with elevated skin pH levels. Following anti-microbiome treatment, the burden of *S. aureus* was found to decrease, corresponding with a reduction in disease severity [32,33].

The identification of biomarkers in AD enhances the understanding of its pathogenesis, facilitates better characterization of AD patients, and allows for the customization of therapy for individuals. Among the various biomarkers, predictive markers (which indicate response to treatment) and prognostic markers (which assess disease progression) are of particular interest. Recent studies have demonstrated that baseline expression levels of the chemokine C-C motif ligand 22 (CCL22) at AD lesions serve as a reliable predictive biomarker for responses to topical crisaborole, cyclosporine, and fezakinumab. Additionally, baseline levels of C-X-C motif chemokine ligand 2 (CXCL2), a cytokine associated with TH17 cells, have been shown to predict response to dupilumab treatment [34]. Regarding prognostic biomarkers, several factors have been identified, including abnormal lipid composition in the stratum corneum and thymus and activation-regulated chemokine (TARC)/CCL17 levels. Elevated serum IgE levels are also potentially associated with the onset of AD [35]. Dysregulation of immune markers differs between children and adults. In infants with AD, there is an increase in the number of circulating regulatory T cells, accompanied by elevated expression of Th17-related proteins, including IL-17A, IL-17F, and PI3, as well as IFN-γ [36]. Additionally, Th2-related proteins, such as IL-4, CCL13, and CCL17, are upregulated in infant AD, with a further increase in Th2-related proteins observed in adolescents and adults with AD [37]. In contrast, the function of the Th1 axis (characterized by IFN-γ, CXCL9, CXCL10, and CCL2) is diminished in infants with AD and gradually recovers from childhood to adulthood [36,37]. Furthermore, elevated levels of coagulation and diabetes-related cardiovascular proteins are observed exclusively in adult patients with AD [37].

Interestingly, AD may influence other dermatological conditions, including chronic hand eczema and contact dermatitis. Chronic hand eczema is a highly refractory inflammatory skin disorder. A study employing tape stripping and high-throughput RNA sequencing has demonstrated that in the presence of AD, chronic hand eczema is more likely to exhibit a type 2 inflammatory response, characterized by the critical involvement of IL-13 and CCL17. Genetic biomarkers associated with this condition show a significant correlation with clinical severity. Conversely, in the absence of AD, chronic hand eczema tends to present a type 1 inflammatory pattern [38]. The study has elucidated the relationship between chronic hand eczema and AD, highlighting subtype-specific therapeutic targets. Furthermore, another study has elucidated the relationship between contact dermatitis and AD, revealing that patients with AD exhibit diminished and altered contact hypersensitivity responses to common allergens. This is attributed to baseline immune abnormality, including a notable reduction in TH1 cytokine production, increases in TH17 cytokines, and inconsistent upregulation of TH2 cytokines [39]. These findings indicate that patients with AD demonstrate an overall weakened immune response and a modified immune cell profile when exposed to allergens.

## 3. Clinical Features

AD is a long-term condition marked by episodes of acute inflammation and scratching, alternating with periods of symptom relief. Key symptoms include intense itching, skin redness, and dryness, with itching being the most common complaint. This itching often intensifies at night and is aggravated by allergens, low humidity, sweating, and various skin irritants [40]. The intensity of itching inversely correlates with quality of life, significantly increasing the disease burden on affected individuals.

**Figure 2:**
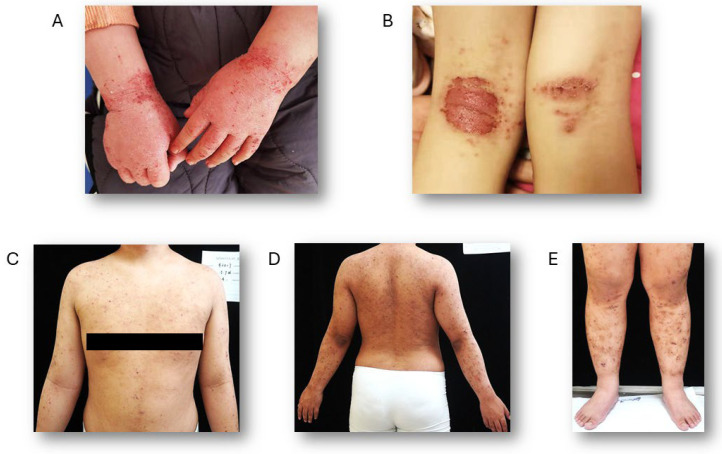
Representative images of AD. (**A**) A 4-year-old child presenting with AD on the hands. (**B**) A 14-year-old child exhibiting exudative AD on the flexor surfaces of the lower limbs. (**C**–**E**) A 32-year-old man with AD affecting both the upper trunk and lower limbs.

The occurrence of skin fissures increases the possibility of infections, especially from *Staphylococcus aureus*, which may result in symptoms like oozing and crusting [41]. AD can manifest in specific skin regions based on the patient’s age, including flexural and extensor surfaces, as well as the face and neck. In infants, lesions primarily occur on the cheeks and the extensor surfaces of the trunk and limbs, while the central face and diaper area are generally spared. For children aged 2 to 12 years, the flexural areas of the upper and lower limbs are commonly affected, along with the eyelids and neck. In adults, typical areas of involvement include the face, neck, and upper trunk, with chronic cases often impacting the hands and leading to lichenification of the skin [42] (Figure 2).

AD is clinically categorized into two subtypes based on IgE levels and allergy history: extrinsic (EAD) and intrinsic (IAD). EAD is defined by elevated total IgE (>200 kU/L), the presence of allergen-specific IgE, and a personal or family history of allergic conditions. In contrast, IAD presents with normal total IgE levels (≤200 kU/L) and no evidence of allergen-specific IgE or a personal/family allergic history. Despite sharing similar clinical symptoms, the two subtypes have distinct underlying pathophysiologies [43,44].

## 4. Diagnosis

A diagnosis of AD is established based on clinical presentation and patient history, with the exclusion of various erythematous and eczematous conditions. One of the earliest and most established diagnostic frameworks is the 1980 criteria developed by Hanifin and Rajka, which requires that patients meet three out of four major criteria and three out of twenty-three minor criteria [45,46] (Table 1). However, these criteria can be overly complex for clinical applications. In response, a consensus conference in 2003, led by the American Academy of Dermatology (AAD), proposed revised Hanifin and Rajka criteria that are more streamlined and applicable across all age groups affected by AD [46,47] (Table 2). The updated criteria differentiate between essential elements required for diagnosis, such as pruritus; significant characteristics that bolster the diagnosis, such as early onset; and associated features that indicate the diagnosis but are nonspecific, such as lichenification [41,46,47]. The UK Working Party criteria provide a straightforward and accessible method for diagnosing AD. To receive a diagnosis, a patient must present with an itchy skin condition and at least three of the following criteria: a history of involvement of the skin flexures, a history of asthma or hay fever, a history of generalized dry skin within the past year, the onset of the rash before the age of two, or observable flexural dermatitis [48]. Physicians are advised to examine the flexural surfaces, hands, face, and neck for signs of AD. In infants, seborrheic dermatitis may be mistaken for AD. Additionally, various conditions must be ruled out prior to diagnosing AD, including allergic reactions, fungal irritations, contact dermatitis, psoriasis, and others [47,49,50,51,52,53,54,55,56,57] (Table 3).

## 5. Disease Severity and Clinical Outcome Assessments

Following the diagnosis of AD, it is essential to assess the severity of the disease. To date, twenty-eight different scales have been identified, but no single gold standard has emerged [47]. These scales utilize various methodologies, including grid patterns, objective disease characteristics, and extent, while some also incorporate subjective features.

The most commonly employed disease severity scales include the Scoring Atopic Dermatitis (SCORAD) index, the Eczema Area and Severity Index (EASI), Investigator’s Global Assessment (IGA), and the Six Area, Six Sign Atopic Dermatitis (SASSAD) severity score [58,59]. AAD recommends the SCORAD index, EASI score, and Patient-Oriented Eczema Measure (POEM) severity scale, as these have been adequately tested and validated for measuring disease severity and clinical outcomes, making them suitable for clinical practice [47] (Table 4). In addition to the aforementioned scoring systems, several other tools have been developed and utilized in clinical settings in recent years, including the Atopic Dermatitis Control Tool (ADCT) and the Peak Pruritus Numerical Rating Scale (PP-NRS). The ADCT is a self-reported questionnaire specifically designed to assess the control of AD. It comprises six domains: overall disease severity, onset of intense itching, bothersome intensity, sleep disturbances, daily life disruptions, and emotional disturbances [60]. Due to its brief completion time, the ADCT is convenient for clinical practice, making it an effective tool for both physicians and patients to monitor the control of the disease. PP-NRS is a tool designed to assess the intensity of the “worst itch” experienced by patients over the past 24 h, utilizing a scale ranging from 0 (indicating no itch) to 10 (representing the worst itch imaginable). This tool facilitates the monitoring of patient responses to current treatments. A reduction of 2 to 4 points on the scale is indicative of clinical improvement [61].

## 6. Current Management and Treatment

### 6.1. Non-Pharmacological Management

Non-pharmaceutical interventions constitute the first-line management strategy for AD. As xerosis is a cardinal feature of the condition, regular application of moisturizers is indicated to maintain cutaneous hydration. Optimal moisturizing formulations contain emollients, occlusive agents, and/or humectants, which function to reduce trans-epidermal water loss and enhance water retention within the skin barrier [62]. The most recent 2024 guidelines from the AAD provide additional recommendations concerning dilute bleach baths and elimination diets. Dilute bleach baths are recommended for patients with moderate-to-severe AD, primarily for their antibacterial efficacy against Staphylococcus aureus overgrowth. A sodium hypochlorite concentration of 5% to 8.25% is advised, with the specific dilution contingent upon bathtub volume [63]. Conversely, elimination diets receive a conditional recommendation due to the low-certainty evidence for their efficacy and a concomitant potential risk of nutritional deficiency [63,64].

### 6.2. Topical Treatments

For patients refractory to non-pharmacological management, escalation to topical pharmacotherapy is indicated. This therapeutic class encompasses prescription-grade moisturizers (regulated as medical devices), topical corticosteroids (TCS), topical calcineurin inhibitors (TCIs), topical phosphodiesterase-4 inhibitors (PDE4is), topical Janus kinase inhibitors (JAKi), and topical antimicrobials [63].

As the cornerstone of AD management across all age groups, TCS are the first-line standard of care for acute flare mitigation [65]. Agent selection necessitates careful consideration of potency and vehicle formulation, tailored to the patient’s age, anatomical site(s) involved, and required treatment duration. Clinicians must be cognizant of potential adverse effects associated with prolonged use, including local cutaneous reactions such as atrophy and dyspigmentation, as well as a potential increased risk of glaucoma or cataracts with peri-ocular application [17,65].

TCIs, including pimecrolimus 1% cream and tacrolimus (0.03% and 0.1%) ointment, constitute a secondary class of anti-inflammatory agents. They are indicated for maintenance therapy and relapse prevention following initial control with TCS. TCIs are particularly suited for application on sensitive areas (e.g., face, anogenital, intertriginous regions), on skin exhibiting corticosteroid-induced atrophy, and for scenarios requiring avoidance of chronic TCS use. A frequently reported adverse effect is transient application-site irritation, which often subsides with continued use [15,62,63,65].

Crisaborole, a PDE4i, exerts its mechanism by modulating intracellular cyclic adenosine monophosphate (cAMP) levels, resulting in the downregulation of pro-inflammatory cytokines, including TNF-α, and T-cell mediators such as IL-2 [66]. Formulated as a 2% topical ointment, it provides a non-steroidal alternative for mild-to-moderate AD [67]. Adverse effects are predominantly mild-to-moderate, transient application-site reactions, including pain, burning, and pruritus, which rarely necessitate therapy discontinuation [55]. Another PDE4i. namely difamilast, targets PDE4B subtype specifically. It was first approved in Japan to treat both pediatric and adult AD patients. Although its underlying mechanisms in treating AD has not been fully understood, its safety and efficacy in reducing AD symptoms, including inflammation, itch, skin barrier dysfunction, have been tested in various clinical trials [68].

JAKi represents an emerging therapeutic class that targets the JAK-STAT pathway, critically involved in Th2-mediated cytokine signaling (e.g., IL-4, IL-13, IL-31). Novel topical agents include ruxolitinib (a JAK1/2 inhibitor) and delgocitinib (a pan-JAK inhibitor). In phase 3 trials, ruxolitinib cream showed considerable effectiveness for mild-to-moderate atopic dermatitis, achieving all primary and secondary endpoints while maintaining a positive safety profile. Delgocitinib ointment has received approval in Japan for both pediatric and adult populations with atopic dermatitis. Frequently reported side effects include nasopharyngitis, upper respiratory infections, and headaches [69].

The utility of other topical agents, such as antihistamines, coal tar derivatives, and non-specific phosphodiesterase inhibitors, is limited by insufficient efficacy data and a higher burden of adverse effects, confining their use primarily to investigational contexts [62]. A recent clinical trial has confirmed the therapeutic efficacy of a novel topical agent, tapinarof, in the treatment of AD. Tapinarof is a nonsteroidal topical aryl hydrocarbon receptor (AhR) agonist that was previously utilized in patients with plaque psoriasis [70]. Last year, the FDA approved its use for children over two years of age and adults with AD. This agent has been shown to rapidly alleviate AD-related itch with mild and tolerable side effects [70]. However, a limitation of tapinarof is the lack of long-term safety and efficacy evaluations.

### 6.3. Systemic Therapy

Systemic therapeutic intervention is indicated for patients with moderate-to-severe AD refractory to optimized topical management. This treatment modality encompasses several classes of agents, including biologic therapies—notably monoclonal antibodies that inhibit key cytokine pathways such as IL-4/IL-13 signaling or specifically target IL-13, small molecule immunosuppressants and ultraviolet phototherapy [63].

#### 6.3.1. Biologics

**Figure 3:**
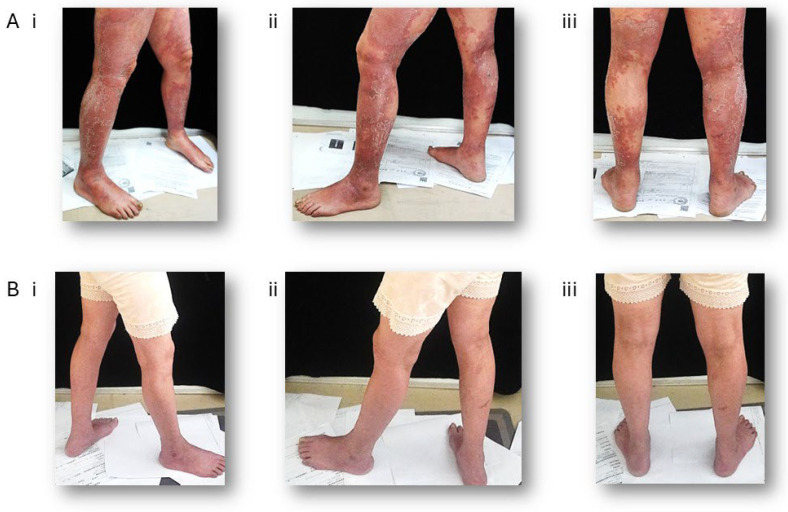
Clinical improvement in a patient with severe AD following Dupilumab treatment. A 65-year-old woman has been given 300 mg Dupilumab by subcutaneous injection every other week for 40 weeks. (**A**) Representative photographs of the lower limbs at baseline (prior to treatment). (**B**) Corresponding photographs taken after treatment. (i) right view, (ii) left view, (iii) posterior view.

Dupilumab was first granted FDA approval in April 2017 for the treatment of moderate-to-severe atopic dermatitis in adults whose condition is not sufficiently managed by topical prescription therapies or when these treatments are not suitable [71]. Its mechanism of action involves the blockade of the shared IL-4 and IL-13 receptor subunit, thereby inhibiting downstream JAK-STAT pathway signaling. This inhibition directly counteracts three central pathophysiological mechanisms of AD: the impairment of skin barrier function, immunoglobulin class switching to IgE, and Th2 cell differentiation [72]. Dupilumab has demonstrated significant efficacy and a favorable safety profile across Phase I to III clinical trials. Additionally, dupilumab has been demonstrated to be safe for use in children aged 6 months to less than 6 years, as evidenced by a phase III clinical trial. It is well tolerated and exhibits an acceptable safety profile [73]. The recommended dosing schedule includes an initial subcutaneous loading dose of 600 mg, followed by a maintenance dose of 300 mg given subcutaneously every two weeks. Its safety profile is better than that of traditional systemic immunosuppressants such as cyclosporine or methotrexate, with injection-site reactions and conjunctivitis being the most commonly reported side effects [72,74] (Figure 3).

Tralokinumab (marketed as Adbry™ or Adtralza^®^) is a human IgG4 monoclonal antibody that specifically neutralizes IL-13 by inhibiting its binding to the IL-13Rα1/IL-4Rα receptor complex. It is recommended as an effective and usually well-tolerated treatment option for adult patients with moderate-to-severe AD who qualify for systemic therapy [75]. Additionally, it has also showed efficacious and well tolerated in adolescents aging 12–17 [76]. Furthermore, a clinical study has demonstrated a long-term disease control effect for up to four years [77]. The dosing schedule consists of an initial subcutaneous loading dose of 600 mg, administered as four injections of 150 mg each, followed by a maintenance dose of 300 mg, given every two weeks as two 150 mg injections. Tralokinumab can be used alone or in conjunction with topical corticosteroids [78]. The most common adverse event associated with its use is conjunctivitis, which was reported in 8% of patients receiving tralokinumab compared to 3% of those on placebo in clinical trials. The majority of these cases were mild to moderate in severity and resolved during the treatment period [78,79]. Another new monoclonal antibody targeting IL-13 is lebrikizumab. It has received approval from both the FDA and European Medicines Agency (EMA) for the treatment of moderate-to-severe AD in adults and adolescents aged over 12 years with a body weight of at least 40 kg, serving as a systemic agent. Clinical studies have demonstrated that lebrikizumab acts quickly to reduce AD-related itch and sleep disturbances, significantly improving patients’ quality of life [80].

In addition to the currently approved biologics, several novel monoclonal antibodies targeting distinct immunological pathways are under investigation in clinical trials for atopic AD. The OX40–OX40 ligand (OX40L) costimulatory pathway plays a critical role in T-cell activation, differentiation, and the formation of immunological memory. Therapeutic inhibition of the OX40–OX40L interaction represents a promising strategy, potentially offering both short-term disease control and long-term modulation of the immune response for sustained clinical benefit. Several investigational agents targeting this pathway—including telazorlimab, rocatinlimab, amlitelimab, and IMG007—are currently being evaluated in clinical trials for AD [15,81].

Beyond OX40 inhibition, other monoclonal antibodies directed against key cytokines and alarmins involved in AD pathogenesis have also demonstrated positive preliminary results in clinical studies. These include agents that block the activity of IL-5, IL-12, IL-23, TSLP, and IL-22, highlighting the diverse and expanding landscape of targeted immunomodulatory therapies for this disease [15,] (Figure 1).

#### 6.3.2. Small Molecules

As previously noted in the context of topical therapy, JAKi also constitute a class of systemic treatments for AD. Currently, three oral JAKi—baricitinib, abrocitinib, and upadacitinib—are approved by FDA and the EMA for patients with moderate-to-severe AD. Baricitinib is a selective inhibitor of JAK1 and JAK2. It is suggested for AD patients who are not well-response to TCS or systemic treatments, such as ciclosporin, or not suitable for these therapies. It can be used alone or with TCS, reducing disease severity significantly and rapidly. Furthermore, it provides an oral option for AD patients who are suggested for subcutaneous biologics [82]. As second-generation agents, abrocitinib and upadacitinib exhibit enhanced selectivity for JAK1 [83]. A network meta-analyses comparing different systemic immunosuppressants has shown that 200 mg abrocitinib and 30 mg upadacitinib can be more effective than dupilumab and baricitinib as second-line therapies. 30 and 15 mg upadacitinib are more effective than ciclosporin A as a first-line therapy. In adolescents, Upadacitinib 15 mg, abrocitinib 200 and 100 mg can be more effective than dupilumab [84]. However, as small molecule agents with systemic bioavailability, the potential for adverse effects is a significant consideration. In 2021, the FDA mandated a class-wide Boxed Warning for all JAKi regarding the risk of serious infections, malignancy, major adverse cardiovascular events (MACE), thrombosis, and mortality. This decision was based on safety data from a post-marketing trial of tofacitinib in patients with rheumatoid arthritis, which demonstrated an increased risk of venous thromboembolism (VTE) [85]. Although the reported incidence of VTE in AD populations is relatively low (approximately 0.2–0.3 events per 100 patient-years), a thorough assessment of individual VTE risk factors is imperative prior to initiation [86]. Other serious risks requiring monitoring include malignancy and renal or hepatic impairment. In clinical practice, the most frequently reported adverse events are gastrointestinal disturbances (e.g., abdominal pain, diarrhea, nausea, vomiting), acne, and headache [87]. Despite this adverse event profile, oral JAK inhibitors are generally well-tolerated by most patients.

Beyond JAKi, conventional immunosuppressants represent a cornerstone in the therapeutic arsenal for moderate-to-severe AD, particularly in cases refractory to first-line treatments. This class includes azathioprine, cyclosporine, methotrexate, and mycophenolate mofetil. Azathioprine is a synthetic purine analogue and prodrug for 6-mercaptopurine. Its mechanism of action is that of an antimetabolite; it is incorporated into DNA and RNA, thereby inhibiting purine synthesis and suppressing the proliferation of rapidly dividing cells, with a particular affinity for lymphocytes. Notably, it demonstrates a more potent suppressive effect on T-cell-mediated immunity than on B-cell function [88]. Cyclosporine, a calcineurin inhibitor, acts as a potent suppressor of T-cell activation by inhibiting calcineurin-mediated cytokine transcription. In the context of AD, its efficacy is attributed to the reduction of key pathogenic cytokines derived from Th2 (e.g., IL-13, CCL17), Th22 (e.g., IL-22, S100As), and Th17 pathways. This broad cytokine suppression subsequently modulates the epidermal hyperplasia and aberrant differentiation characteristic of AD lesions [89]. Methotrexate is an antifolate agent whose immunosuppressive effects are mediated through multiple pathways. Its primary mechanism involves the inhibition of dihydrofolate reductase, leading to impaired DNA synthesis and the suppression of cellular proliferation. This action ultimately results in the downregulation of activated immune cells involved in the AD inflammatory cascade [90]. Mycophenolate mofetil (MMF) functions as a selective, non-competitive inhibitor of the type II isoform of inosine monophosphate dehydrogenase (IMPDH), a key enzyme in the de novo pathway of guanine nucleotide synthesis. By depleting intracellular guanine nucleotides, MMF preferentially inhibits the proliferation of T and B lymphocytes, which are highly dependent on this pathway. Although its primary licensed indication is in transplant rejection prophylaxis, MMF is frequently utilized off-label for the management of refractory AD [91].

#### 6.3.3. Phototherapy

Phototherapy is a well-established second-line intervention for AD when first-line topical and conventional systemic therapies are ineffective, contraindicated, or poorly tolerated. According to the AAD guidelines, narrowband ultraviolet B (NB-UVB) phototherapy is recommended for patients with moderate-to-severe AD who are refractory to, or have contraindications for, mid- to high-potency topical corticosteroids and systemic agents, including biologics [63]. The therapeutic mechanism of NB-UVB is multifactorial. It induces the formation of DNA photoproducts, which disrupt transcription and inhibit the rapid cell division characteristic of hyperproliferative epidermis. Its immunomodulatory effects are extensive, including a reduction in the cutaneous T-cell population and an enhancement of T-regulatory cell activity. Furthermore, the generation of oxidative stress impedes antigen-presenting function and dendritic cell-mediated T-cell activation. These combined actions result in a downstream shift in cytokine profile, characterized by a suppression of pro-inflammatory cytokines and an upregulation of anti-inflammatory mediators such as IL-10 [92].

The 2024 AAD guidelines provide a prioritized framework for systemic therapy in AD. These guidelines provide strong endorsements for the use of dupilumab, tralokinumab, abrocitinib, baricitinib, and upadacitinib. They also offer conditional support for phototherapy, azathioprine, cyclosporine, methotrexate, and mycophenolate mofetil. In contrast, the guidelines recommend against the regular use of systemic corticosteroids [93].

## 7. Limitations and Future Perspectives

AD is characterized by a complex and multifaceted clinical presentation, driven by a pathophysiology involving numerous overlapping immunological, genetic, and environmental factors. This review has focused on elucidating the pathophysiology and contemporary management strategies of AD. Several limitations of this work must be acknowledged. First, the section on management primarily introduces the most widely utilized Western pharmacological agents. It does not encompass discussions of complementary and alternative therapies, such as traditional Chinese medicine or herbal-based treatments, which warrant further systematic investigation to evaluate their efficacy and integration into clinical practice. Second, the pathophysiological mechanisms and treatment paradigms discussed are predominantly relevant to the adult population. The review does not address the unique considerations required for special populations, including infants, children, and pregnant or lactating individuals. A dedicated and comprehensive review of AD management in these distinct demographic groups is therefore indicated. Third, the clinical guidelines referenced in this review are predominantly those published by the AAD. To provide a more global perspective, future iterations should incorporate and critically appraise guidelines from other international bodies, such as those from European, Asian, and Chinese dermatology associations.

Looking ahead, advancing the understanding and management of AD necessitates targeted future research. Priorities include conducting long-term outcome studies for existing and emerging treatment modalities across diverse patient populations. Given the strong genetic predisposition underlying AD, the development of personalized medicine approaches, tailored to an individual’s specific genetic and molecular profile, holds significant promise for optimizing therapeutic efficacy. Furthermore, the application of nanotechnology, particularly the use of nanoparticle-based drug delivery systems, represents a promising frontier. Such systems offer the potential to enhance therapeutic efficacy through targeted delivery, reduce systemic adverse effects, improve skin penetration, and allow for more precise dosing control.

## 8. Conclusions

In summary, the management of AD presents a multifaceted challenge that requires a comprehensive understanding of its pathophysiology, varied treatment options, and individualized patient care. While non-pharmacological strategies and topical therapies remain foundational in the initial management of the condition, systemic therapies, including biologics and phototherapy, are crucial for patients with moderate to severe forms of AD who do not respond to conventional treatments.

Emerging therapies continue to expand the therapeutic landscape, offering hope for improved outcomes. However, ongoing research is essential to evaluate the long-term efficacy and safety of these treatments, address existing knowledge gaps, and refine management strategies. By fostering collaboration among dermatologists, researchers, and patients, the field can advance toward more effective and personalized care for individuals living with atopic dermatitis. Ultimately, a holistic approach that incorporates both medical and psychosocial support will enhance the quality of life for patients affected by this chronic condition.

## Figures and Tables

**Table 1:** Hanifin and Rajka criteria for diagnosis of AD [45,46].

**Major criteria** (at least **3** must be met)
1. Pruritus
2. Typical morphology and distribution
Adults: Flexural lichenification
Infancy: Facial and extensor involvement
3. Chronic or chronically relapsing dermatitis
4. Personal or family history of atopic disease (asthma, allergic rhinitis, AD)
**Minor criteria** (at least **3** must be met)
1. Xerosis
2. Ichthyosis/hyperlinear palms/keratosis pilaris
3. Immediate skin test reactivity
4. Elevated serum IgE
5. Early age of onset
6. Tendency for cutaneous infections
7. Tendency to nonspecific hand/foot dermatitis
8. Nipple eczema
9. Cheilitis
10. Recurrent conjunctivitis
11. Dennie-Morgan infraorbital folds
12. Keratoconus
13. Anterior subscapsular cataracts
14. Orbital darkening
15. Facial pallor/facial erythema
16. Pityriasis alba
17. Anterior neck folds
18. Pruritus when sweating
19. Intolerance to wool and lipid solvents
20. Perifollicular accentuation
21. Food hypersensitivity
22. Course influenced by environmental and/or emotional factors
23. White dermatographism or delayed blanch to cholinergic agent

**Table 2:** American Academy of Dermatology Diagnostic Criteria for Atopic Dermatitis [46,47].

**Essential features (must be present)**
Pruritus
Eczema (acute, subacute, chronic)
Typical morphology and age-specific patterns *
Chronic or relapsing history
**Important features (seen in most cases, adding support to the diagnosis)**
Early age of onset
Atopy
Personal and/or family history
IgE reactivity
Xerosis
**Associated features (suggest the diagnosis, but not for defining or detecting AD)**
Atypical vascular responses (e.g., facial pallor, while dermographism, delayed blanch response)
Keratosis pilaris/pityriasis alba/hyperlinear palms/ichthyosis
Ocular/periorbital changes
Other regional findings (e.g., perioral changes/periauricular lesions)
Perifollicular accentuation/lichenification/prurigo lesions
**Exclusionary conditions**
Scabies
Seborrheic dermatitis
Contact dermatitis
Ichthyoses
Cutaneous T-cell lymphoma
Psoriasis
Photosensitivity dermatoses
Immune deficiency diseases
Erythroderma of other causes

* Patterns include: 1. Facial, neck, and extensor involvement in infants and children; 2. Current or prior flexural lesions in ang age group; 3. Sparing of groin and axillary regions.

**Table 3:** Differential diagnosis of AD [47,49,50,51,52,53,54,55,56,57].

**Diagnosis**	**Main Features**	**Ref.**
Scabies	Increased nocturnal pruritus, a small, short (3–7 mm) and linear-to-serpiginous burrow visible in the skin surface caused by scabies mites	[49]
Seborrheic dermatitis	Tend to coexist with AD in infants, salmon-colored papules and greasy scale crust in scalp and face	[50]
Contact dermatitis	Shown in the exposure area to allergens or irritants. Acute: oedema, erythema and vesicles; chronic: xerosis (dry skin), scales, hyperkeratosis and fissures	[51]
Ichthyoses	Impaired keratinocyte differentiation and abnormal formation of the epidermal barrier result in itching, frequent infections, reduced sweating (hypohidrosis) accompanied by heat intolerance, as well as various complications related to vision, hearing, and nutrition	[52]
Cutaneous T-cell lymphoma	Erythematous, dry patches, skin biopsy, PCR and other laboratory tests are needed for diagnosis	[53]
Psoriasis	Immune-mediated erythematous patches with silvery scale, nail can be affected	[54]
Photosensitivity dermatoses	Cutaneous eruptions when exposed to ultraviolet or visible radiation can be induced by drugs	[55]
Immune deficiency diseases	AD can be skin manifestation; genetic testing is needed	[56]
Erythroderma of other causes	Skin inflammatory state, with associated skin barrier and metabolic dysfunctions, clinical history, biopsies and other tests are needed	[57]

**Table 4:** Scoring systems for assessment AD severity [58,59].

**Scoring System**	**Parameters**	**Severity Rating**	**Ref.**
SCORAD	six signs—erythema, excoriation, swelling, oozing/crusting, lichenification and dryness	Clear (0–9.9)	[58]
		Mild (10.0–28.9)	
	on eight body sites, and pruritus and sleeplessness	Moderate (29.0–48.9)	
		Severe (49.0–103)	
EASI	four signs—erythema, excoriation, swelling and lichenification	Clear (0)	
		Almost clear (0.1–1.0)	
		Mild (1.1–7)	
	on four body sites	Moderate (7.1–21)	
		Severe (21.1–50)	
		Very severe (50.1–72)	
IGA	FDA categorization of AD severity based on the investigator’s subjective assessment of a representative lesion	0 = clear	
		1 = almost clear	
		2 = mild	
		3 = moderate	
		4 = severe	
SASSAD	six signs (erythema, exudation, excoriation, dryness, cracking and lichenification	0 = absent	[59]
		1 = mild	
	on six areas (head and neck, trunk, hands, arms, legs and feet	2 = moderate	
		3 = severe	
POEM	seven symptoms scored over past week (itch, sleep, bleeding, weeping/oozing, cracking, flaking, and dryness/roughness)	Clear/almost clear (0–2)	
		Mild (3–7)	
		Moderate (8–16)	
		Severe (17–24)	
		Very severe (25–28)	

## Data Availability

No new data were created or analyzed in this study. Data sharing is not applicable to this article.

## Abbreviations

The following abbreviations are used in this manuscript:

AD	atopic dermatitis
AAD	American Academy of Dermatology
AhR	aryl hydrocarbon receptor
cAMP	cyclic adenosine monophosphate
CCL	chemokine C-C motif ligand
CXCL	C-X-C motif chemokine ligand 2
DCs	dendritic cells
EAD	extrinsic atopic dermatitis
EASI	Eczema Area and Severity Index
EMA	European Medicines Agency
FLG	filaggrin gene
IAD	intrinsic atopic dermatitis
IFN	interferon
IGA	Investigator’s Global Assessment
IL	interleukins
IMPDH	inosine monophosphate dehydrogenase
JAKi	Janus kinase inhibitors
MACE	major adverse cardiovascular events
MMF	Mycophenolate mofetil
NB-UVB	narrowband ultraviolet B
OVOL	OVOL ovo like transcriptional repressor
OX40L	OX40–OX40 ligand
PDE4is	phosphodiesterase-4 inhibitors
PM	particulate matter
POEM	Patient-Oriented Eczema Measure
SASSAD	Six Area, Six Sign Atopic Dermatitis
SCORAD	Scoring Atopic Dermatitis
SPINK5	spinous layer protein 5
TCIs	topical calcineurin inhibitors
TCS	topical corticosteroids
Th	T helper
TLA4	T-lymphocyte-associated protein 4
TNF-α	tumor necrosis factor alpha
TSLP	thymic stromal lymphopoietin
VTE	venous thromboembolism
